# Coral-zooxanthellae meta-transcriptomics reveals integrated response to pollutant stress

**DOI:** 10.1186/1471-2164-15-591

**Published:** 2014-07-12

**Authors:** Kurt A Gust, Fares Z Najar, Tanwir Habib, Guilherme R Lotufo, Alan M Piggot, Bruce W Fouke, Jennifer G Laird, Mitchell S Wilbanks, Arun Rawat, Karl J Indest, Bruce A Roe, Edward J Perkins

**Affiliations:** Environmental Laboratory, US Army Engineer Research and Development Center, Vicksburg, MS 39180 USA; Advanced Center for Genome Technology, University of Oklahoma, Norman, OK 73019 USA; Badger Technical Services, San Antonio, TX 71286 USA; Department of Geology, Urbana-Champaign, University of Illinois, Urbana-Champaign, IL 31801 USA; Division of Marine Geology and Geophysics, University of Miami, Miami, FL 33149 USA; Institute for Genomic Biology, Urbana-Champaign, University of Illinois, Illinois, IL 31801 USA; Translational Genomics Research Institute, Phoenix, AZ 85004 USA

**Keywords:** Coral holobiont, Marine pollution, Meta-transcriptomics, *Acropora*, Zooxanthellae, RDX, Symbiosis, Transcriptional network, Next generation sequencing

## Abstract

**Background:**

Corals represent symbiotic meta-organisms that require harmonization among the coral animal, photosynthetic zooxanthellae and associated microbes to survive environmental stresses. We investigated integrated-responses among coral and zooxanthellae in the scleractinian coral *Acropora formosa* in response to an emerging marine pollutant, the munitions constituent, 1,3,5-trinitro-1,3,5 triazine (RDX; 5 day exposures to 0 (control), 0.5, 0.9, 1.8, 3.7, and 7.2 mg/L, measured in seawater).

**Results:**

RDX accumulated readily in coral soft tissues with bioconcentration factors ranging from 1.1 to 1.5. Next-generation sequencing of a normalized meta-transcriptomic library developed for the eukaryotic components of the *A. formosa* coral holobiont was leveraged to conduct microarray-based global transcript expression analysis of integrated coral/zooxanthellae responses to the RDX exposure. Total differentially expressed transcripts (DET) increased with increasing RDX exposure concentrations as did the proportion of zooxanthellae DET relative to the coral animal. Transcriptional responses in the coral demonstrated higher sensitivity to RDX compared to zooxanthellae where increased expression of gene transcripts coding xenobiotic detoxification mechanisms (i.e. cytochrome P450 and UDP glucuronosyltransferase 2 family) were initiated at the lowest exposure concentration. Increased expression of these detoxification mechanisms was sustained at higher RDX concentrations as well as production of a physical barrier to exposure through a 40% increase in mucocyte density at the maximum RDX exposure. At and above the 1.8 mg/L exposure concentration, DET coding for genes involved in central energy metabolism, including photosynthesis, glycolysis and electron-transport functions, were decreased in zooxanthellae although preliminary data indicated that zooxanthellae densities were not affected. In contrast, significantly increased transcript expression for genes involved in cellular energy production including glycolysis and electron-transport pathways was observed in the coral animal.

**Conclusions:**

Transcriptional network analysis for central energy metabolism demonstrated highly correlated responses to RDX among the coral animal and zooxanthellae indicative of potential compensatory responses to lost photosynthetic potential within the holobiont. These observations underscore the potential for complex integrated responses to RDX exposure among species comprising the coral holobiont and highlight the need to understand holobiont-species interactions to accurately assess pollutant impacts.

**Electronic supplementary material:**

The online version of this article (doi:10.1186/1471-2164-15-591) contains supplementary material, which is available to authorized users.

## Background

Coral reef ecosystems are experiencing dramatic worldwide declines due to the coupled impacts of local habitat degradation and global warming [1–3]. As these threats mount, the survival of corals increasingly depends on their capacity to rapidly establish physiological acclimation to environmental change [4–7]. While corals have shown remarkable resilience to extreme environmental changes through geological time [8], relatively little is known of the mechanisms that have facilitated acclimation.

Environmental change in the form of pollutant release into marine environments also poses potential threats to coral survival. Historic and on-going military activities have resulted in the presence of discarded and unexploded ordinance (UXOs) containing the high explosive 1,3,5-trinitro-1,3,5 triazine (RDX) in critical coral reef habitats (i.e. Vieques, Puerto Rico [9] and Oahu, Hawaii [10]) posing unknown threats to already embattled ecosystems as the UXOs age and potentially leach RDX. Therefore, understanding the response of coral to ordinance compound exposures such as RDX is critical for the conservation and preservation of these reef ecosystems.

Corals represent a complex community called the “coral holobiont” composed of the coral animal, algal symbionts (*zooxanthellae*) within the coral tissues, and microbes inhabiting the coral mucus [11–14]. The photosynthetic activity of the zooxanthellae has been observed to contribute as much as 95% of the carbon requirement for daily respiration and growth of the coral holobiont [15]. In return, the corals provide a protective environment for the zooxanthellae in addition to essential nutrients derived from heterotrophic feeding [15, 16] and fixation by associated microbes [17].

As a critical step toward deciphering changes in coral holobiont interactions that reflect acclimation to environmental stressors, we have completed the first broad-scale meta-transcriptomics study of the coral *Acropora formosa*. In marked contrast to previous sequencing efforts where coral genomic DNA was physically separated from that of zooxanthellae before analysis (i.e. [18–21]), we have characterized the protein-coding genes for the *in situ* holobiont including both coral and zooxanthellae (the *metatranscriptome*). This approach provided the ability to investigate global transcript expression within an intact symbiotic relationship between coral and zooxanthellae and thus enable the observation of interactive metabolism among species. *A. formosa* represents a useful coral model species that can be easily cultivated for use in controlled laboratory experimentation. Furthermore, *A. formosa* is ecologically and physiologically similar to other ecologically relevant *Acropora* species occurring in reef tracts around the world [22].

Here, we examined coral holobiont responses to RDX exposure using bioconcentration measures, coral-zooxanthellae meta-transcriptomics, transcriptional network analysis and preliminary studies of zooxanthellae and mucocyte density. In addition to providing meta-transcriptome characterization for the eukaryotic components of the *A. formosa* coral holobiont, this study demonstrates the integrative responses among the coral animal and zooxanthellae that comprise the coral holobiont to an emerging marine pollutant.

## Methods

### Ethics statement

The work described in this paper represents laboratory studies conducted using aquarium-cultured *Acropora formosa* coral fragments that were purchased from the Oceans, Reefs and Aquaria Company (ORA, Harbor Branch Oceanographic Institution, Ft. Pierce, FL). The study did not involve vertebrate testing or experiments with threatened or endangered species.

### RDX exposures

Fragments of the branched coral *A. formosa* ranging in size from 4 to 7 cm were allowed to acclimate to exposure chambers for 24 hours prior to experiment initiation (details on shipping and processing of corals is provided in the Additional file 1). Exposure chambers consisted of 38 L glass aquaria filled with 20 L of reconstituted seawater (Crystal Sea® Marinemix, Marine Enterprises International, Baltimore, MD) and equipped with a Coralife Super Skimmer and Bio Balls (Central Garden and Pet Co., Walnut Creek, CA) placed in the outlet filtration box. Given facility limitations and the requirement of this specific exposure chamber setup to maintain coral health, the experimental design included single exposure chambers containing five independent biological replicates. While single exposure chambers do not allow exclusion of variation in responses due to the exposure apparatus versus RDX exposure concentration, our principle concern was variation in biological responses at the individual level. Water quality, analytical chemistry and environmental conditions (methods and results described below) were closely monitored for consistency across all chambers to ensure variations in responses were due to the RDX exposure concentrations. We considered the individual coral fragments within each tank to represent true statistical replicates as has been described previously [23–25] only after careful empirical consideration through monitoring criteria contributing to statistical independence [26] and establishing dose–response relationships across the independent exposure aquaria (see Additional file 1 for detailed discussion).

Exposures included a control chamber (0 mg/L) and RDX-exposure chambers where RDX dissolved directly in seawater was added to exposure media targeting 0.5, 1.0, 2.0, 4.0 and 8.0 mg/L concentrations (see Additional file 1 for comments on expected exposure levels in the environment). Exposure media were maintained at 27°C by water re-circulating REMCOR heating/cooling units (REMCOR Products Company, Glendale Heights, IL). Salinity was maintained at 32 ppt and a 16:8 hr light-to-dark photoperiod applied with four high-intensity full-spectrum Phillips, Alto Collection F40T12/DX, 40 Watt, 6500 Kelvin, 48 inch light bulbs (Phillips Corporation, Amsterdam, Netherlands) housed in high reflectance Lithonia Lighting® Model 1241DP chromed light fixtures (Acuity Brands Company, Conyers, GA) placed approximately 7 cm above each exposure chamber. RDX concentrations and water quality parameters including temperature, salinity, pH, dissolved oxygen concentration, ammonia concentration, and nitrogen concentration were measured daily. At the termination of the RDX exposure bioassay, 3 of the 5 coral fragments from the control as well as each of the RDX exposure conditions were selected at random and immediately fixed in RNA Later™ (Ambion, Austin, TX) following manufacturers recommendations. For the remaining coral fragments, one was selected at random for RDX bioaccumulation analysis in coral soft tissues, while the final fragment was used for preliminary investigations of RDX effects on coral soft tissue histology/histochemistry.

### Analytical chemistry of exposure water

The concentration of RDX in exposure chambers was determined by daily sampling of exposure water. Two mL of exposure water was pipetted into 4 mL amber HPLC sampler vials and analyzed following USEPA method 8330 [27] on a Waters HPLC using C18 and CN columns (detailed description provided in the Additional file 1). All RDX results are from the Supelco C18 column for which there were no interferences. The laboratory reporting limit for all analytes was 0.5 μmol/L (~0.1 mg/L) for water samples.

### Normalized cDNA library construction

RNA extractions were conducted by gently scraping the coral soft tissue from the calcium carbonate skeleton and then following the Qiagen, RNeasy® Mini RNA extraction kit protocol (Qaigen Inc. Valencia, CA). The quantity and quality of the RNA extracted from each tissue sample was confirmed as described in Gust et al. [28]. The *A. formosa* RNA library was assembled by pooling all RNA samples from every replicate sampled in both control and all RDX exposure treatments. Reverse transcription of full length eukaryotic cDNAs was accomplished using Clontech SMART-PCR (Clonetech Laboratories Inc. Mountain View, CA). Due to the use of poly(d)T primers in this process, prokaryotic sequences which lack poly(A)-tails were excluded from the coral holobiont library. In order to generate sequence information for as many unique cDNAs as possible, the cDNA library was normalized using the Trimmer cDNA Normalization Kit (Evrogen JSC, Moscow, Russia) to insure sequencing of both high and low abundance transcripts.

### cDNA library sequencing and annotation

The normalized cDNA library was sequenced using Roche 454 GS-FLX pyrosequencing [29] with several modifications that improved sequence reproducibility while reducing labor-intensive sample manipulations [30, 31]. Flowgrams from the GS-FLX were assembled by Newbler Assembler (Roche Applied Science). Contiguous sequences (contigs) and singletons were searched against the NCBI Genbank protein database using blastx where E ≤ 10^-5^ was considered a significant match to an expressed sequence tag (EST). Gene targets were annotated where possible with identity and functionality using Kyoto Encyclopedia of Genes and Genomes (KEGG, http://www.genome.jp/kegg/) and EuKaryotic Orthologous Groups (KOG, http://genome.jgi.doe.gov/Tutorial/tutorial/kog.html) databases.

### Phylogenetic characterization of the coral holobiont meta-transcriptome

Phylogenetic designations connected to best blastx sequence matches were generated for all contigs and singletons having significant blastx scores (E ≤ 10^-5^). These phylogenetic identities were manually searched against published literature and assigned to one of three taxonomic groups (1) coral, (2) algal symbiont or (3) other. The group represented as “other” consisted of sequences where the phylogenetic origin was not representative of the two major eukaryotic groups contributing to the coral holobiont (i.e. fungi), or could not be definitively assigned to only one of these groups (i.e. various protists). All matches to prokaryotes were removed from the eukaryotic library. A “strict taxonomy” and an “inclusive taxonomy” were developed given our phylogenetic inquiry. The strict taxonomy only included associations beginning at the phylogenetic class anthozoa to represent coral and algal symbionts only included *Symbiodinium* species as well as algal species recognized to have direct associations with coral, all based on primary literature search. For the inclusive taxonomy, all matches to animal species were included within the coral taxon and all plant and alga species were included within the algal symbiont taxon. Finally, given that the meta-transcriptomic library for the coral holobiont was normalized, we tested if the relative expression of unique transcripts between the two eukaryotic contributors to the holobiont (coral and algal symbionts) was proportional using a binomial test (SAS v.9.2, SAS Inc. Raleigh, NC).

### Development of custom microarray

The 7,500 sequences identified with the lowest E values (which represent the best similarity to known genes) were uploaded to eArray (Agilent Technologies, Palo Alto, CA) in both forward and reverse sequence orientation (due to lack of reading-frame information to assess the “sense” strand). eArray was utilized to incorporate these 15,000, 60mer oligonucleotide probes into a custom microarray design (Agilent Technologies) for the eukaryotic members of the *A. formosa* holobiont. This design represents 7,500 sense-antisense probe-pairs providing quality control of the microarray analysis for cross hybridization. Specifically, one probe in each probe pair represents a nonsense sequence and our expectation was that no target should have specific binding to it (see below for anti-sense strand exclusion methods). The phylogenetic characterization of transcriptome sequence information (see previous section) was leveraged to provide taxonomic characterizations for all probes on the microarray. The custom microarrays were developed for the *A. formosa* holobiont prior to the removal of sequences with blastx matches to prokaryotes, therefore 5.8% of microarray probes represent non-quality assured sequence matches. Expression data for these probes was removed prior to final microarray analysis.

### Microarray hybridizations

The effects of RDX exposure on transcript expression in the eukaryotic components of the holobiont (coral animal and algal symbiont) were assessed simultaneously by investigating quality-assured RNA [32] collected from whole corals exposed in the exposure bioassay. The microarray hybridization experiment included 3 biological replicates for each of the following conditions: control, 0.5, 2, and 8 mg/L, RDX (target concentrations). Microarray hybridizations were conducted utilizing the Agilent QuickAmp, One-Color Microarray Hybridization protocol (Agilent Technologies) following manufacturer’s instructions.

### Microarray analysis

An Axon GenePix® 4000B Microarray Scanner (Molecular Devices Inc., Sunnyvale, CA) was used to scan microarrays at 5 μm resolution. Data were extracted from microarray images using Agilent Feature Extraction software, version 9.5.1 (Agilent Technologies). Microarray data were normalized to the 50th percentile within each array followed by median scaling among all exposures using GeneSpring Software version 7.3 (Agilent Technologies). GeneSpring was additionally used to conduct statistical analyses to identify differentially expressed transcripts (DET) among treatments using one-way ANOVA including Benjamini and Hochberg multiple testing corrections [33]. A post-hoc test including a parametric t-test (p = 0.05) and log_2_ fold change cutoff of ≥ 1.5 was used to discern statistically significant differences in transcript expression for each RDX treatment relative to the control. Given the sense-antisense architecture of the microarray (see above), all differentially expressed microarray targets were examined to identify and eliminate microarray targets for which both the sense and antisense probes were differentially expressed. Microarray data are publicly available at the Gene Expression Omnibus (GEO, http://www.ncbi.nlm.nih.gov/geo/info/linking.html) under series accession GSE27624.

### Delineating meta-transcriptomic expression in response to RDX exposure

A Chi square test (Sigma Stat, v.3.1.1, Systat Software, Inc Chicago, IL) was used to test for differences in the proportion of transcripts differentially expressed among the phylogenetic groups (coral, algal symbiont and “other”) across RDX-exposure concentrations. Chi-square tests were also used to determine if the number of differentially expressed transcripts changed in response to RDX concentration for each phylogenetic group. Similarly, we tested if the proportion of DETs changed among phylogenetic groups when investigating the number of DETs relative to the total number of probes represented for each group on the microarray. Finally, binomial tests (SAS v.9.2, SAS Inc. Raleigh, NC) were conducted to determine if the proportion of significant transcripts that had increased versus decreased expression deviated from the proportion expected by chance (i.e. 1:1 ratio).

An investigation of transcript functions affected by RDX exposure among the phylogenetic groups was also conducted. Specifically, KOG, a eukaryotic representation of the Clusters of Orthologous Groups (COG) database [34], identities for all differentially expressed transcripts were leveraged to determine which metabolic pathways represented the primary functional targets impacted by RDX exposure.

### Reverse engineering of transcriptional network

We used an ordinary differential equations-based method described by Lai et al. [35] to construct an integrated transcriptional network. The network included all DET from both coral and zooxanthellae to assess correlations in expression within and among species for the most highly-represented KOG functions. A visual representation of the resulting network was generated using Cytoscape [36].

### Reverse-transcriptase, quantitative polymerase chain reaction (RT-qPCR)

Microarray results were validated by RT-qPCR for 13 unique transcript targets found to be of critical importance for the interpretation of RDX effects in the coral holobiont in addition to one regulatory “control” transcript, “actin gene” for *Acropora millepora* (Additional file 2: Table S1). Transcript expression levels were examined using DNase (Qiagen, Valencia, CA) treated total RNA from each of the three biological replicates used in microarray hybridizations (see Additional file 1 for methods). RT-qPCR data was analyzed with SDS 2.2 software package (Applied Biosystems, Foster City, CA) using the ΔΔCT method to quantify results as recommended by the developer. Assumptions inherent in the ΔΔCT analysis were observed to be met sufficiently insuring accuracy of downstream analysis. Actin is recognized to have relatively stable expression independent of external stimuli in cnidarians [37]. One-way ANOVA on threshold cycle (Ct) values including control, 0.5, 1.8 and 7.2 mg/L exposures indicated that results are consistent with the assumption that actin expression remained consistent across treatments (p = 0.849), and was therefore selected as the internal normalizer for relative quantification (RQ). The characteristics of actin expression included low variability in relative quantification (RQ) and Ct value near the median of all RT-qPCR reactions. Fold change values (log_2_) were calculated using RQ results where values represent transcript expression in RDX-treated coral relative to control coral. The 95% confidence interval (95% C.I.) was calculated around the mean relative expression for each RDX treatment. Confidence intervals that did not include unity were considered differentially expressed relative to controls as described in Rawat et al. [38].

### Analytical chemistry of RDX-tissue residues

One coral fragment per treatment was sampled for body residue determination. The soft coral tissues were gently scraped from each *A. formosa* fragment with a sterile scalpel and transferred to QBiogene - Lysing Matrix A 2.0 ml tubes (MP Biomedicals LLC) containing garnet matrix and one 0.6 cm ceramic sphere. HPLC-grade acetonitrile (0.25 ml, Sigma-Adrich) was added to each tube. The tissue samples were weighed and then homogenized in a FAST Prep-24 mini-bead-beater instrument (MP Biomedicals, Santa Ana, CA) for one minute and centrifuged at 7500 g for three minutes. After centrifugation, 0.1 mL of the extract was removed and mixed with 0.1 mL of CaCl_2_ (5 g/L). Subsamples were added to 0.15 mL glass inserts and placed into 4 mL amber HPLC sampler vials. The samples were analyzed following USEPA method 8330 [27] on a Waters HPLC using C18 and CN columns (detailed description provided in the Additional file 1). All RDX results are from the Supelco C18 column for which there were no interferences. Because RDX body residue was measured in only one replicate coral per treatment, statistical comparisons were not conducted. The laboratory reporting limit for all analytes was 5 μmol/kg (~1 mg/kg) for tissue samples.

### Preliminary study of coral tissue histology and histochemistry

Changes in the coral tissue density of mucocytes and symbiotic zooxanthellae in response to RDX exposure was investigated using ultra-high resolution analysis of coral holobiont histochemistry. Three technical replicate cuttings from a single coral fragment were collected from each exposure condition and fixed in Carnoy’s solution (60% ethanol, 30% chloroform and 10% glacial acetic acid). Methods for CaCO_3_ skeleton demineralization, tissue embedding, histological tissue sectioning, histochemical staining, image acquisition and analysis are described in Piggot et al. [39]. Briefly, histological sections were individually imaged for fluorescence with mucocyte cells represented by *N*-acetylglucosamine content bound by wheat germ agglutinin-conjugated Alexa Fluor 647 and zooxanthellae cells represented by chlorophyll auto-fluorescence induced with a metal halide bulb emitting through a HQ480/50 excitation filter. Mucocyte and zooxanthellae densities were quantified as described in Piggot et al. [39] with means and standard deviations calculated for control and the 8 mg/L (target concentration) RDX exposure treatment. The standard deviation represented the technical variation within a single replicate.

## Results

### Exposure conditions

The concentration of RDX in the water remained relatively stable during the 5-d exposure (Additional file 2: Table S2a) where mean measured exposure concentrations were 0.50, 0.93, 1.77, 3.67 and 7.18 mg/L. Measured concentrations are used to represent RDX treatments throughout the remaining text. The greatest decrease in concentration between days 0 and 5 was 9%, observed in the 1 mg/L treatment. All water quality parameters were within adequate range according to guidance in ASTM [40], (Additional file 2: Table S2b).

### Coral meta-transcriptome sequencing, assembly and annotation

The sequencing effort yielded 144.61 megabases in 702,750 reads. After removal of adapter sequences, the assembled reads yielded 61,691 contiguous sequences (contigs) and 127,925 singletons. Contigs and singletons were searched against the NCBI Genbank protein database using blastx identifying 12,141 significant gene matches at E ≤ 10^-5^ after removal of microbial sequences (Additional file 2: Table S3). Gene targets were annotated where possible with identity, functionality, gene ontology, KEGG orthology and KEGG pathway information.

### Phylogenetic characterization of the coral meta-transcriptome

**Figure 1:**
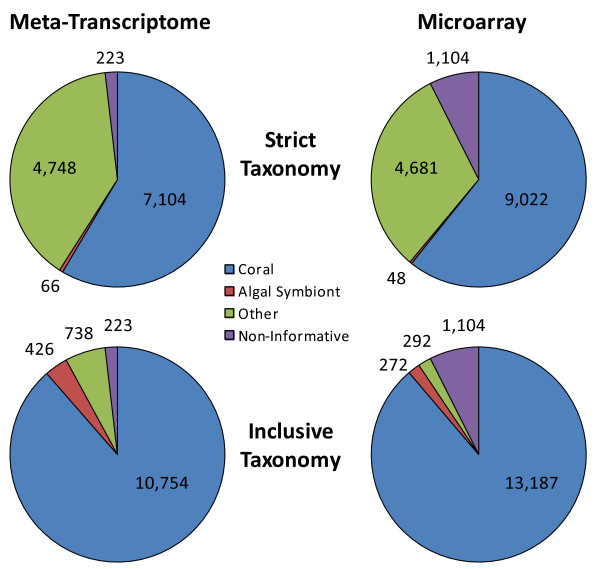
**Taxonomy of meta-transcriptome sequence data generated for the** ***Acropora formosa*** **coral animal and associated zooxanthellae.** Taxonomies are provided for the meta-transcriptome as well as for the probes represented on the *A. formosa* meta-transcriptome microarray. A strict and inclusive taxonomy are provided based on criteria explained in the Methods.

Examination of the strict taxonomy generated for the coral meta-transcriptome indicated that more transcripts could be definitively ascribed to coral as compared to algal symbionts (P < 0.001, Figure 1, Additional file 2: Table S3). The inclusive taxonomy provided a similar result where most transcripts were matched to coral (P < 0.001), however the inclusive taxonomy provided a broader representation of transcripts with lineages likely related to algae symbionts (Figure 1). The taxonomic distribution of transcripts represented as probes on the *A. formosa* holobiont microarray largely matched the distribution observed within the meta-transcriptome (Figure 1, Additional file 2: Table S4). The taxonomic distribution served as a reference point for investigating the relative contribution of the coral animal and the algal symbionts regarding transcript expression discussed below.

Although only 70 transcripts had best blastx matches to the *Acropora* genus, 6,943 best matches were made with *Nematostella vectensis* (Additional file 2: Table S3), a sea anemone with a fully sequenced genome [41] to which *A. formosa* is related at the phylogenetic class anthozoa. Matches to zooxanthellae included 10 sequences matching *Amphidinium carterae* and 56 matching species of the genus *Symbiodinium* which are the primary photosynthetic endosymbionts of Acroprid corals [42]. The majority of these matches were to the *Symbiodinium* clade 3. Expanding upon the strict taxonomy to an inclusive taxonomy (Additional file 2: Table S3) allowed for vastly increased functional associations to the two predominant eukaryotic classes of the coral holobiont community (corals and symbiotic algae) and provided a novel perspective for understanding dynamics within the holobiont in response to the RDX stressor exposure.

### RDX-tissue residues

**Figure 2:**
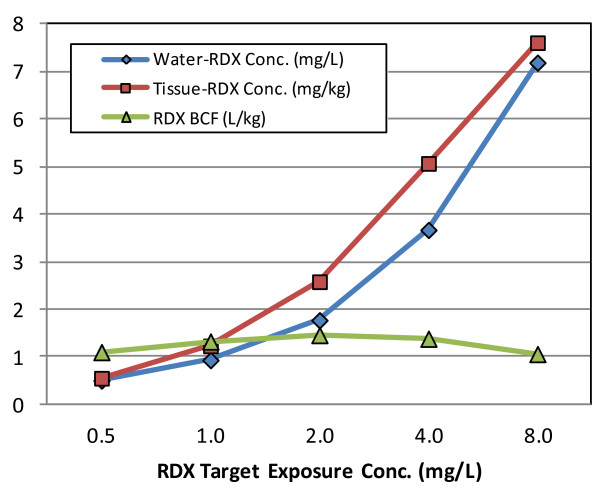
**Measured RDX concentrations in exposure chambers and in coral tissue.** Additionally, the bioconcentration factor (BCF) for RDX is provided. The y-axes for tissue residues and BCF are mg/kg and L/kg, respectively.

The concentration of RDX in coral tissues increased with increasing exposure concentration (Figure 2). The bioconcentration factor (BCF) values for RDX were relatively similar among treatments, with no apparent relationship with the RDX concentration in water (Figure 2; mean BCF = 1.3). The mean BCF for *A. formosa* exposed to RDX was within the range of values observed for a variety of aquatic invertebrates and fish [43].

### Microarray results overview

**Figure 3:**
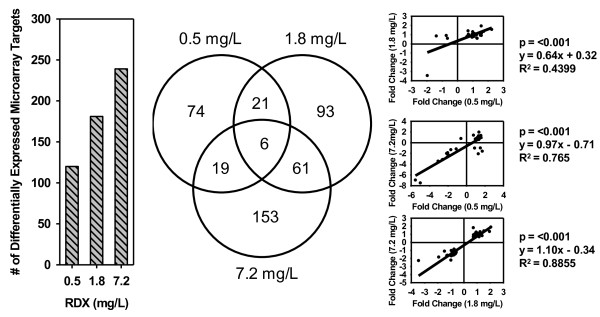
**Effect of RDX on transcript expression in** ***Acropora formosa*** **.** The bar chart and Venn diagram represent the total number of transcripts observed to have undergone significant differential expression compared to controls. The Venn diagram displays the number of differentially expressed transcripts that were common among the RDX treatments. Regression plots show log_2_ fold-change relationships among differentially expressed transcripts found in common among RDX treatments.

As an initial quality control step, a total of 14, 4 and 36 microarray targets within the 0.5, 1.8 and 7.2 mg/L RDX treatments, respectively, were identified to be differentially expressed in both the sense and antisense microarray probes when searched across all differentially expressed transcripts (DET). These targets represented non-specific probes and were therefore eliminated from the overall results set. The summation of the DET within each RDX treatment indicated a positive relationship between RDX-exposure concentration and the number of DET (Figure 3) yielding a total of 120, 181 and 239 DET in the 0.5, 1.8 and 7.2 mg/L RDX treatments, respectively (all differentially-expressed targets presented in Additional file 2: Table S5). The greatest degree of commonality in DET was found between the 1.8 and 7.2 mg/L RDX treatments (25.5%), followed by the 0.5 and 1.8 mg/L treatments (14.9%) whereas the least commonality (10.5%) was found among the most disparate RDX-exposure concentrations, 0.5 and 7.2 mg/L (Figure 3). Regression of log_2_ fold-change values for DET that were common among RDX treatments showed significant positive correlations (Figure 3). This indicates that within the DET there is a trend toward conservation of transcriptomic responses to RDX exposure across >1 order of magnitude difference in RDX concentration.

### Differential impact of RDX among coral animal and zooxanthellae

**Figure 4:**
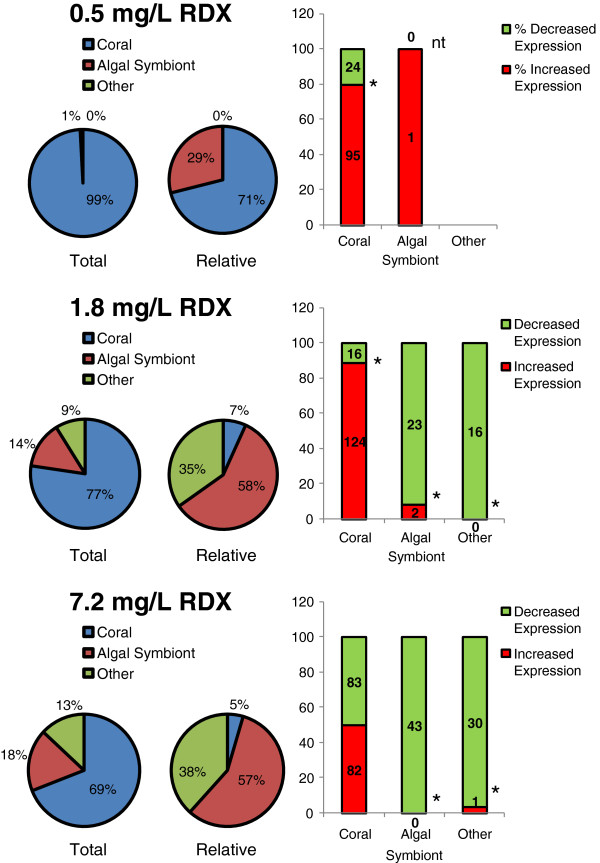
**Effect of RDX concentration on transcript expression in the coral and zooxanthellae.** The pie chart labelled “Total” provides the percent of the total suite of transcripts that had significant differential expression summed across coral, algal symbionts, and other (“other”, representing unconfirmed taxonomic classifications). The pie chart labelled “Relative” provides the relative percentage of transcripts that had significant differential expression normalized to the total number of targets present for each taxonomic class on the microarray (See Additional file 2: Table S4 for target distribution on microarray). The bar graphs provide the proportion of significant transcripts that had increased vs decreased expression (totals inset in bars). Binomial tests were used to identify differences in the proportions of transcripts having increased : decreased expression with significant difference (p = 0.05) marked with “*” and tests without sufficient data marked with “nt” representing no test.

The proportion of total DET contributed among the coral animal and algal symbionts changed significantly across RDX-exposure treatments (p <0.001) where the relative contribution of algal symbionts increased with increasing RDX concentration (Figure 4). When normalized by the total number of probes represented for each taxonomic group on the microarray, the relative contribution of algae to DET was 58% and 57% in the 1.8 and 7.2 mg/L treatments, respectively (Figure 4). Multiple significant differences in the proportion of transcripts having increased versus decreased expression were observed in response to RDX exposure concentration for coral and the algal symbionts (Figure 4). Coral had a greatly increased proportion of transcripts having increased expression at the 0.5 mg/L (p <0.001) and the 1.8 mg/L (p <0.001) RDX exposures, but not at 7.2 mg/L (p = 1.0). Finally, the proportion of algal symbionts having decreased expression tended to increase with increasing RDX concentrations (Figure 4).

### Impacts of RDX on molecular pathways

Investigation of KEGG functional terms for DET indicated the potential for impacts on a variety of molecular pathways in the RDX exposures (Table 1, and Additional file 2: Table S6). The predominant impact of RDX involved pathways underlying “metabolism”. Specifically, carbohydrate metabolism had the greatest number of total DET summing across all RDX exposure concentrations (Table 1). Differential expression of transcripts involved in amino acid metabolism was another prominent effect observed across RDX treatments. Additional prominent “metabolism” pathways affected by RDX included: energy metabolism, lipid metabolism and metabolism of cofactors and vitamins. Aside from impacts on metabolism, impacts of RDX exposure on pathways involved in “environmental information processing” and “cellular processes” were additionally represented across all RDX treatments.

**Table 1:** **Kyoto Encyclopedia of Genes and Genomes (KEGG) terms associated with transcripts that had significant differential expression in response to RDX exposure compared to controls in a 5d experiment**

0.5 mg/L RDX	Cor	Alg	Oth	1.8 mg/L RDX (cont.)	Cor	Alg	Oth	7.2 mg/L RDX (cont.)	Cor	Alg	Oth
***1. Metabolism***	***14***	***0***	***0***	Pyruvate metabolism	0	0	1	***1.4 Nucleotide Metabolism***	***1***	***1***	***0***
**1.1 Carbohydrate Metabolism**	**5**	**0**	**0**	Glyoxylate and dicarboxylate metabolism	1	0	0	Purine metabolism	1	1	0
Glycolysis/Gluconeogenesis	1	0	0	Butanoate metabolism	0	0	1	***1.5 Amino Acid Metabolism***	***4***	***0***	***3***
Citrate cycle (TCA cycle)	2	0	0	***1.2 Energy Metabolism***	***2***	***1***	***0***	Alanine and aspartate metabolism	0	0	1
Pyruvate metabolism	2	0	0	Oxidative phosphorylation	2	1	0	Glycine, serine and threonine metabolism	2	0	1
***1.2 Energy Metabolism***	***1***	***0***	***0***	***1.3 Lipid Metabolism***	***0***	***0***	***3***	Valine, leucine and isoleucine biosynthesis	1	0	1
Nitrogen metabolism	1	0	0	Fatty acid metabolism	0	0	1	Lysine degradation	1	0	0
***1.3 Lipid Metabolism***	***2***	***0***	***0***	Bile acid biosynthesis	0	0	1	***1.6 Metabolism of Other Amino Acids***	***2***	***0***	***0***
Fatty acid metabolism	1	0	0	Glycerolipid metabolism	0	0	1	Cyanoamino acid metabolism	1	0	0
Arachidonic acid metabolism	1	0	0	***1.5 Amino Acid Metabolism***	***3***	***0***	***3***	Glutathione metabolism	1	0	0
***1.4 Nucleotide Metabolism***	***1***	***0***	***0***	Glutamate metabolism	2	0	0	***1.9 Metabolism of Cofactors and Vitamins***	***7***	***1***	***0***
Purine metabolism	1	0	0	Alanine and aspartate metabolism	0	0	1	One carbon pool by folate	1	0	0
***1.5 Amino Acid Metabolism***	***1***	***0***	***0***	Glycine, serine and threonine metabolism	0	0	1	Porphyrin and chlorophyll metabolism	0	1	0
Glutamate metabolism	1	0	0	Valine, leucine and isoleucine biosynthesis	0	0	1	Limonene and pinene degradation	1	0	0
***1.6 Metabolism of Other Amino Acids***	***2***	***0***	***0***	Arginine and proline metabolism	1	0	0	Phenylpropanoid biosynthesis	1	0	0
Glutathione metabolism	2	0	0	1.6 Metabolism of Other Amino Acids	1	0	0	Streptomycin biosynthesis	1	0	0
***1.7 Glycan Biosynthesis and Metabolism***	***2***	***0***	***0***	Glutathione metabolism	1	0	0	Gamma-Hexachlorocyclohexane degradation	1	0	0
Chondroitin sulfate biosynthesis	1	0	0					Naphthalene and anthracene degradation	1	0	0
Glycan structures - biosynthesis 1	1	0	0	**2. Genetic Information Processing**	**1**	**0**	**1**	Fluorene degradation	1	0	0
				***2.1 Transcription***	***0***	***0***	***1***				
**3. Environmental Information Processing**	**4**	**0**	**0**	Aminoacyl-tRNA biosynthesis	0	0	1	**2. Genetic Information Processing**	**3**	**0**	**2**
***3.1 Membrane Transport***	***1***	***0***	***0***	***2.3 Folding, Sorting and Degradation***	***1***	***0***	***0***	***2.1 Transcription***	***1***	***0***	***1***
ABC transporters - General	1	0	0	Protein export	1	0	0	Aminoacyl-tRNA biosynthesis	1	0	1
***3.2 Signal Transduction***	***3***	***0***	***0***					***2.2 Translation***	***2***	***0***	***1***
MAPK signaling pathway	1	0	0	**3. Environmental Information Processing**	**1**	**1**	**0**	Ribosome	2	0	1
ErbB signaling pathway	1	0	0	***3.2 Signal Transduction***	***1***	***1***	***0***				
Wnt signaling pathway	1	0	0	Two-component system - General	0	1	0	**3. Environmental Information Processing**	**9**	**1**	**0**
				Wnt signaling pathway	1	0	0	***3.1 Membrane Transport***	***1***	***0***	***0***
**4. Cellular Processes**	**6**	**0**	**0**					ABC transporters - General	1	0	0
***4.2 Cell Growth and Death***	***1***	***0***	***0***	**4. Cellular Processes**	**2**	**0**	**0**	***3.2 Signal Transduction***	***8***	***1***	***0***
Cell cycle	1	0	0	***4.3 Cell Communication***	***1***	***0***	***0***	Two-component system - General	0	1	0
***4.3 Cell Communication***	***1***	***0***	***0***	Adherens junction	1	0	0	MAPK signaling pathway	2	0	0
Focal adhesion	1	0	0	Tight junction	1	0	0	ErbB signaling pathway	1	0	0
***4.4 Endocrine System***	***1***	***0***	***0***					Wnt signaling pathway	1	0	0
GnRH signaling pathway	1	0	0	**7.2 mg/L RDX**	**Cor**	**Alg**	**Oth**	VEGF signaling pathway	1	0	0
***4.5 Immune System***	***3***	***0***	***0***	**1. Metabolism**	**26**	**7**	**8**	Calcium signaling pathway	1	0	0
Toll-like receptor signaling pathway	1	0	0	1.1 Carbohydrate Metabolism	6	3	3	Phosphatidylinositol signaling system	2	0	0
T cell receptor signaling pathway	1	0	0	Glycolysis/Gluconeogenesis	1	2	1				
B cell receptor signaling pathway	1	0	0	Pentose and glucuronate interconversions	1	0	0	**4. Cellular Processes**	**6**	**0**	**0**
				Ascorbate and aldarate metabolism	1	0	0	***4.3 Cell Communication***	***3***	***0***	***0***
**5. Human Diseases**	**2**	**0**	**0**	Starch and sucrose metabolism	1	0	0	Focal adhesion	2	0	0
***5.1 Cancers***	***2***	***0***	***0***	Nucleotide sugars metabolism	1	0	0	Tight junction	1	0	0
Colorectal cancer	1	0	0	Pyruvate metabolism	0	1	1	***4.4 Endocrine System***	***1***	***0***	***0***
Renal cell carcinoma	1	0	0	Butanoate metabolism	0	0	1	Melanogenesis	1	0	0
				Inositol phosphate metabolism	1	0	0	***4.5 Immune System***	***1***	***0***	***0***
**1.8 mg/L RDX**	**Cor**	**Alg**	**Oth**	***1.2 Energy Metabolism***	***4***	***2***	***1***	Natural killer cell mediated cytotoxicity	1	0	0
**1. Metabolism**	**13**	**1**	**10**	Oxidative phosphorylation	2	0	1	***4.6 Nervous System***	***1***	***0***	***0***
***1.1 Carbohydrate Metabolism***	***7***	***0***	***4***	Photosynthesis	0	1	0	Long-term potentiation	1	0	0
Glycolysis/Gluconeogenesis	0	0	2	Carbon fixation	0	1	0				
Citrate cycle (TCA cycle)	1	0	0	Methane metabolism	1	0	0	**5. Human Diseases**	**1**	**0**	**0**
Pentose phosphate pathway	1	0	0	Nitrogen metabolism	1	0	0	***5.1 Cancers***	***1***	***0***	***0***
Pentose and glucuronate interconversions	1	0	0	***1.3 Lipid Metabolism***	***2***	***0***	***1***	Glioma	1	0	0
Ascorbate and aldarate metabolism	1	0	0	Fatty acid metabolism	0	0	1				
Starch and sucrose metabolism	1	0	0	Glycerophospholipid metabolism	1	0	0				
Nucleotide sugars metabolism	1	0	0	Arachidonic acid metabolism	1	0	0				

KEGG terms are matched to source sequences: Coral (Cor), Algal Symbionts (Alg), or other (Oth) for non-specific phylogenetic associations.

### Validation of microarray results using RT-qPCR

**Figure 5:**
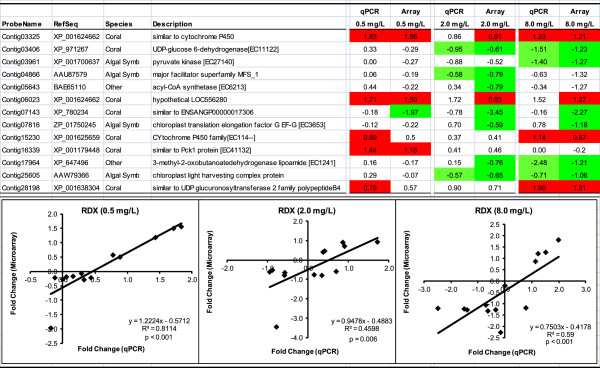
**Comparison of RT-qPCR and microarray results.** Values represent log_2_ fold change in transcript copy number relative to controls. Red and green highlighted cells represent statistically significant increases and decreases in copy number, respectively. Regression analyses represent correlations in log_2_ fold change among microarray and RT-qPCR results. Linear regression significance tests, regression equations and R^2^ values provide evidence of correlations among the gene expression assays. “Species” represent putative source of transcripts. Primer sequences are provided in Additional file 2: Table S1.

Results of the RT-qPCR analysis corresponded directly with microarray results for 80.0%, 53.3% and 73.3% of targets for the 0.5, 1.8 and 7.2 mg/L RDX treatments, respectively (Figure 5). Further, significant positive correlations were observed between transcript expression among RT-qPCR and microarray results (Figure 5) indicating reasonable agreement among analytical techniques. Significant changes in expression were confirmed for a variety of DET representing a broad suite of molecular functions from both coral and symbiotic algae (Figure 5).

### RDX effects on transcriptional networks

**Figure 6:**
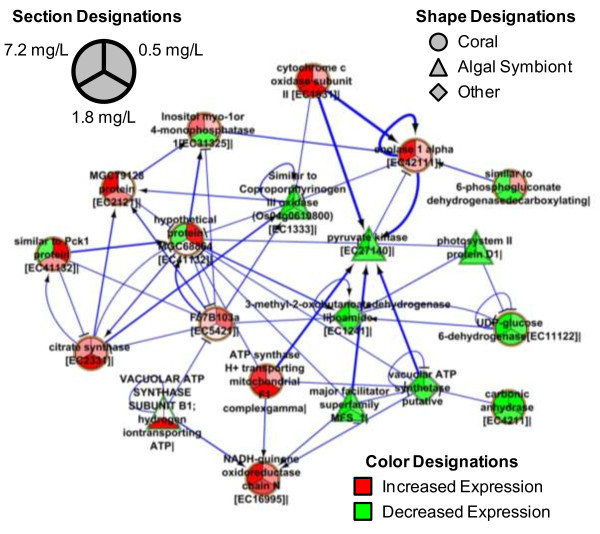
**Transcriptional network inference of coral holobiont interactions.** The network analysis demonstrates correlations of expression among molecular targets and species comprising the coral holobiont in response to RDX exposure. The network includes all Kyoto Encyclopedia of Genes and Genomes terms involved in carbohydrate and energy metabolism. Edge thickness represents correlation strength among nodes. Arrows represent “activation” from one node to the next while “T” ends represent “inhibition”. At least one RDX exposure concentration elicited significant differential transcript expression for each gene, however we have provided expression information for all treatment levels to show trends.

The combined transcriptional network for coral and zooxanthellae generated using DET from the most prominently represented KOG terms, carbohydrate and energy metabolism, indicated several instances of highly correlated expression among the coral animal and the zooxanthellae in response to RDX exposure (Figure 6). Given the intimate bioenergetic connection between coral and zooxanthellae where photosynthesis by zooxanthellae provides as much as 95% of energy resources to the coral holobiont [15], strong correlations in expression within the combined network was expected. Examples of highly correlated expression among species included: pyruvate kinase (XP_001700637) in zooxanthellae correlated with coral expression of transcripts involved in glycolysis (enolase 1 alpha, XP_001632906) and the electron transport chain (ATP synthase H^+^ transporting mitochondrial F1 complex gamma, XP_001638994 and cytochrome C oxidase subunit II, NP_612824). Within the complete transcriptional network, RDX exposure elicited strong interspecies network connections for genes involved in a broad compliment of energy metabolism including: photosynthesis, glycolysis, pyruvate metabolism, the citric acid cycle, electron transport and oxidative phosphorylation (Figure 6, Additional file 2: Table S6). Although these results do not imply direct metabolic connections among these processes, the overall trend of the results indicates a dichotomous response among species where expression of gene transcripts related to nearly all aspects of energy metabolism are decreased in zooxanthellae which is correlated with a broad scale increase in expression of energy metabolism-related gene transcripts in the coral.

### Preliminary study of coral tissue histology and histochemistry

**Figure 7:**
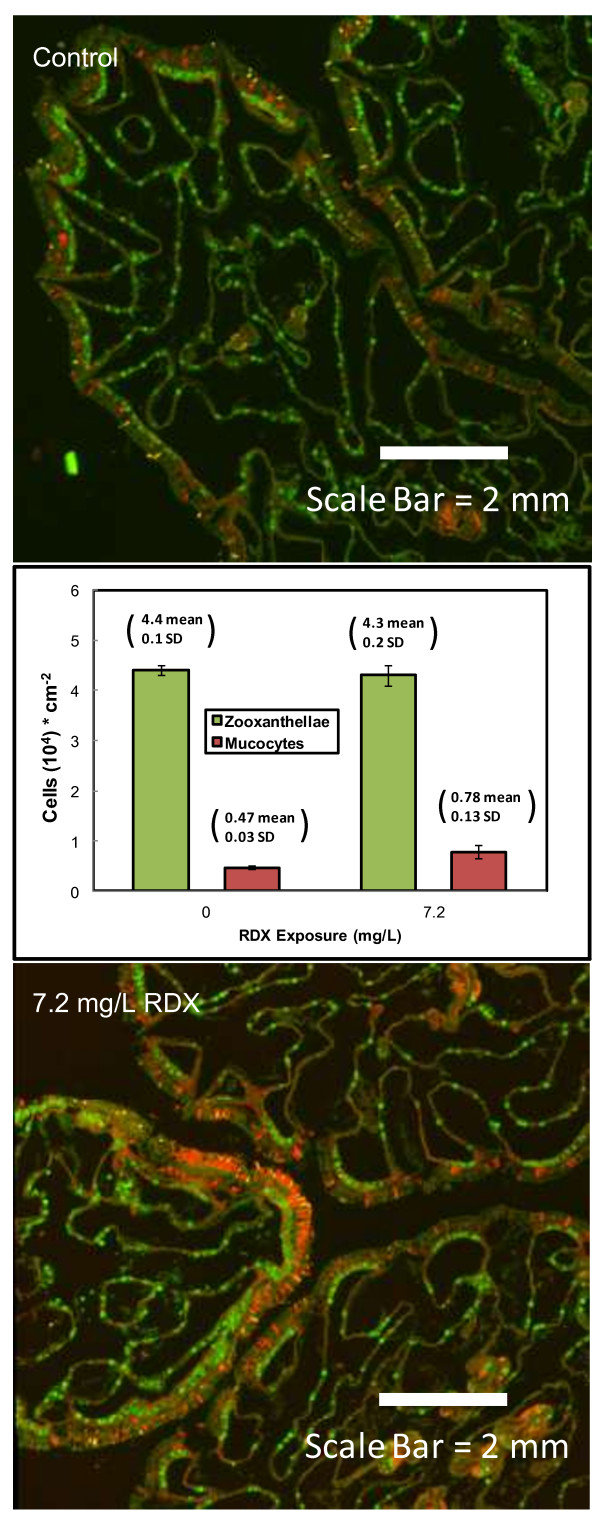
**The effect of RDX exposure on zooxanthellae and mucocyte density in** ***Acropora formosa.*** The bar chart shows the quantification of fluorescence from polyp histology sections showing auto-fluorescent zooxanthellae (green) and WGA-induced fluorescent mucocytes (red).

*A. formosa* exposed to RDX displayed no gross-level visual signs of pigmentation loss or excessive secretion of mucus. Comparison of mucocyte densities between the control (4720 ± 320 cells/cm^2^, mean ± SE) and coral exposed to 7.2 mg/L RDX (7800 ± 1300 cells/cm^2^, mean ± SE) demonstrated the potential for increased mucocyte density at the highest RDX exposure thereby suggesting a possible mucosal protective response by the coral in response to RDX exposure (Figure 7). In contrast, histological examination indicated no observable difference in coral tissue zooxanthellae densities between the control and samples exposed to 7.2 mg/L RDX as the means were similar with relatively little variance in either condition (Figure 7).

## Discussion

The normalized cDNA library for *A. formosa* included transcripts (protein-coding RNAs) for two prominent eukaryotic components of the coral holobiont (the coral animal and symbiotic zooxanthellae), thus representing a meta-transcriptome. Previous large-scale sequencing efforts for coral (*Acropora millepora*, [18, 20] and *Acropora digitifera* [21]) and the close phylogenetic relative sea anemone, *Nematostella vectensis*, [41] were each sequenced without their associated algal symbionts. The inclusion of algal symbionts in our sequencing effort provided the ability to investigate the relative contribution of the coral animal and zooxanthellae to the meta-transcriptome as well as integrated transcript expression.

### The coral holobiont meta-transcriptome

Both the strict and inclusive taxonomies generated for the coral holobiont meta-transcriptome indicated that the overwhelming majority of transcripts originated from coral relative to algal symbionts (Figure 1). We sought to determine if this observation was a result of differences in the number of protein coding genes inherent in the respective genomes of each species. The closest phylogenetic relative of *A. formosa* having a fully sequenced genome, *Acropora digitifera*, was observed to have 23,700 protein coding transcripts [21]. The recently sequenced transcriptome for the primary endosymbiotic dinoflagellate found in corals, *Symbiodinium sp.* reported 40,000-90,000 protein coding genes [44]. These observations compared to our meta-transcriptome results (Figure 1) indicate that contribution to the meta-transcriptome is not proportional to individual species transcriptome size, at least when comparing the eukaryotic contributors to the holobiont. This observation presents questions about individual-species transcriptomic expression as it relates to holobiont function. For example, given the limited percentage of genes attributed to zooxanthellae in the total sequencing pool, is the diversity of transcript expression reduced in zooxanthellae engaged in symbiotic relationships compared to free-living zooxanthellae? Given that the zooxanthellae are engaged in a symbiotic association within the coral animal, division of physiological labor among species may have contributed to a decreased proportional expression of genes within zooxanthellae. For example, if essential nutrients required in zooxanthellae are directly supplied by coral, the need to express the molecular machinery innate in zooxanthellae to capture or create these metabolites is negated, thus the need to express the genes in such pathways is reduced.

### RDX bioaccumulates readily into coral tissue

RDX bioaccumulated into coral tissue in a concentration-dependent manner and exhibited bioconcentration factors (BCF) ranging from 1.09-1.50 L/kg among RDX-exposure concentrations (Figure 2). A BCF near 1.0 L/kg indicates that RDX accumulation in whole-tissue samples mirrors ambient water concentrations, which is consistent with previous observations for RDX [43]. These results indicate that RDX was accumulated in coral tissue and was therefore present to elicit potential toxicological affects within both the coral animal, as well as symbiotic zooxanthellae.

### Differential sensitivity among members of the holobiont at low RDX level

Expression of transcripts in the zooxanthellae was relatively unaffected at the lowest RDX-exposure concentration (0.5 mg/L), whereas a relatively large number of transcripts were differentially expressed in the coral animal (Figure 4). At this concentration, RDX predominantly elicited differential expression of various components of metabolism in the coral animal including carbohydrate, energy, lipid, amino acid and glycan metabolism (Table 1) as well as increased expression of potential detoxification mechanisms (i.e. cytochrome P450, XP_001624662, and UDP glucuronosyltransferase 2 family, XP_001638304, Figure 5).

Carbohydrate metabolism was the most represented second order KEGG term in the 0.5 mg/L treatment. All transcripts involved in carbohydrate metabolism had increased expression (Additional file 2: Table S6) including phosphoenolpyruvate carboxykinase 1 (PCK1, XP_001179448) which was RT-qPCR-validated (Figure 5). PCK1 has been observed to be the regulatory control point in the equilibrium reaction for glycolysis versus gluconeogenesis [45] in a mammalian model, and if this function is conserved in coral, the observed increase in PCK1 expression indicates inertia toward glycolysis. Assuming orthologous gene functions in corals to those observed in the literature, the transcript expression results suggest an equilibrium shift toward increased energy consumption in coral in response to low concentrations of RDX.

Cytochrome P450 metabolism is a key facilitator for chemical detoxification via phase I xenobiotic biotransformation [46] and UDP glucuronosyltransferase 2 represents a xenobiotic phase II metabolism enzyme that conjugates sugar moieties to drugs and endogenous compounds increasing solubility and potential for elimination via excretion [47]. The qPCR validated increase in cytochrome P450 and UDP glucuronosyltransferase 2 family transcriptexpression at this lowest RDX concentration (Figure 5) indicates a likely response to metabolize bioaccumulated RDX. Although the bioaccumulation results suggest these mechanisms were not effective in decreasing RDX body burdens, such responses to chemicals where these detoxification mechanisms metabolize and mitigate toxicity would benefit both coral and the symbiotic zooxanthellae. Regarding the discussion of energy expenditures above, any induced detoxification mechanism requires energy which given the current observations may account for some component of the increased inertia toward energy consumption at the lowest RDX exposure concentration.

### Integrated coral holobiont responses at elevated RDX levels

Similar to the 0.5 mg/L exposure, effects on transcripts coding for genes involved in metabolism were the most pervasive responses at the 1.8 and 7.2 mg/L exposure levels (Table 1). However, in contrast to the coral-dominated response observed at the 0.5 mg/L treatment, the relative proportion of differentially expressed transcripts shifted to a zooxanthellae-dominated response with increasing RDX concentration (Figure 4). The most strongly represented metabolic function was carbohydrate metabolism in each the 1.8 and 7.2 mg/L RDX exposures, with additional marked representation of transcripts involved in energy and amino acid metabolism (Table 1). Cofactor/vitamin metabolism was additionally highly represented in the 7.2 mg/L treatment. Each of these metabolic functions is discussed regarding the integrative response by the coral animals and zooxanthellae in the following.

### Carbohydrate metabolism

Carbonic anhydrase activity has been observed to be required by both species in invertebrate-algal symbiosis to support carbon fixation from CO_2_ via photosynthesis [16] as well as playing a key role in coral skeleton deposition [48]. Transcripts coding for carbonic anhydrase had decreased expression at 7.2 mg/L in coral (AAM94169) and in zooxanthellae (AAW79301, Additional file 2: Table S5) indicating both reduced potential for coral skeleton growth and reduced potential to generate carbon substrates for metabolism via carbohydrate metabolic pathways. Regarding impacts on carbohydrate metabolism, decreased metabolic potential was also demonstrated in zooxanthellae where RT-qPCR validated reductions in pyruvate kinase (XP_001700637), the enzyme that catalyzes the reaction where phospoenolpyruvate is metabolized yielding pyruvate and ATP [49] was observed (Figure 5, Additional file 2: Table S6). These results are suggestive of reduced potential for carbon fixation and cellular energy production via carbohydrate metabolism in the zooxanthellae as well as reduced potential for coral skeleton deposition in the RDX exposure.

Changes in transcript expression related to carbohydrate metabolic pathways in the coral animal also reflected the potential for diminished substrate availability at the 1.8 and 7.2 mg/L RDX exposure concentrations. First, expression of transcripts coding for genes that metabolize large 6-carbon carbohydrate substrates was reduced in coral. For example, RT-qPCR validated reduced expression of UDP-glucose 6-dehydrogenase (XP_971267) was observed with increasing RDX-exposure concentrations (Figure 5, Additional file 2: Table S6). UDP-glucose 6-dehydrogenase catalyzes reactions oxidizing the 6 carbon glucose substrate thereby reducing NAD^+^ to NADH [50] which is a key energy-carrying molecule involved in cellular energy (ATP) production. Additionally, decreased expression of a gene transcript involved in metabolism of a 6 carbon intermediate substrate in the pentose phosphate pathway, 6-phosphogluconate dehydrogenase (XP_001627413, [51]) was observed in the coral animal. In contrast to expression changes related to large 6 carbon carbohydrate substrates, expression of transcripts coding for genes that metabolize small 2- and 3-carbon substrates had increased expression in corals. For example, at the 1.8 mg/L exposure concentration, citrate synthase (XP_001641037), a highly conserved enzyme known to regulate the first reaction in the citric acid cycle converting the 2 carbon substrate acetyl CoA to citrate [52] had increased expression in coral (Additional file 2: Table S6). Additionally, the coral animal had increased transcript expression of enolase 1 alpha (XP_001632906), the enzyme that catalyzes the reaction converting the 3-carbon substrate phosphoglycerate to phospoenolpyruvate at the 7.2 mg/L exposure concentration. These results suggest that in the face of reduced carbon fixation and energy production resulting from RDX exposure in zooxanthellae, larger carbohydrate substrates (i.e. glucose and pentose) may be in short supply causing corals to increase expression of enzymes that can capture energy from intermediate 2 and 3 carbon metabolites to maintain energy budgets.

### Energy metabolism

The observation that RDX exposure may cause diminished carbon fixation as indicated through reduced transcript expression of carbonic anhydrase in both coral and zooxanthellae (described above), it is intuitive that additional impacts on energy metabolism in the holobiont were observed. Reduced expression of transcripts coding photosystem II protein (CAI58622, Additional file 2: Table S6) as well as RT-qPCR validated reductions in pyruvate kinase (XP_001700637), chloroplast light harvesting complex protein (AAW79366) and major facilitator superfamily (AAU87579) in algal symbionts (Figure 5) are additional indicators of reduced potential for photosynthesis. Highly-stressed corals (particularly heat stressed) may eject zooxanthellae resulting in coral bleaching events [4] however, RDX exposure did not affect the density of algal symbionts (zooxanthallae) in coral tissue even at the highest RDX exposure concentration (Figure 7). Therefore, the decrease in transcriptional expression for zooxanthellae-associated genes, including those involved in photosynthesis, was unlikely an artifact of a bleaching event. Photosynthesis by zooxanthellae supports as much as 95% of energy metabolism in the coral holobiont [15]. Increased expression of gene transcripts involved in electron transport chain-based ATP production were observed in the coral animal at both the 1.8 and the 7.2 mg/L RDX treatments likely representing a compensatory response to decreased cellular energy availability stemming from apparent decreased photosynthetic potential in zooxanthellae (Figure 6 and Additional file 2: Table S6). Given these expression results, we hypothesize that RDX may impair photosynthetic activity in zooxanthellae thereby affecting energy availability for the overall coral holobiont. In future studies, measuring photosynthetic rates in the holobiont under RDX exposures could explicitly test this hypothesis.

### Amino acid metabolism

Changes in expression of transcripts involved in amino acid metabolism occurred in the coral animal, but not in zooxanthellae. RT-qPCR validated an inverse relationship between 3-methyl-2-oxobutanoatedehydrogenase lipoamide (XP_647496) transcript expression with increasing RDX-exposure concentration indicating reduced processing of amino acid substrates [53] and thereby reduced ATP consumption to fuel this process. These results are consistent with a metabolic shift to conserve energy resources.

### Metabolism of cofactors and vitamins

Similar to observations at the lowest RDX exposure concentration, increased expression of cytochrome P450 and UDP glucuronosyltransferase 2 family transcripts were observed in the RDX-exposed coral animal and validated using RT-qPCR (Figure 5 and Additional file 2: Table S6). In contrast, no transcriptional effects on cytochrome P450, UDP glucuronosyltransferase 2 family, or other phase I or II detoxification mechanisms were observed in zooxanthellae suggesting coral may be the principle component of the holobiont that actively initiated mechanisms to metabolize and potentially detoxify RDX.

### Mucus production in response to RDX

The preliminary histochemistry assessment suggested that RDX exposure may have the potential to increase coral-derived mucocyte density at the highest RDX-exposure concentration (Figure 7). Corals have been observed to respond to a wide variety of environmental stressors such as crude oil, copper sulphate, mercury, increased sea surface temperature and decreased salinity by excreting copious amounts of mucus (see review in Brown and Bythell [13]). When stimulated, coral tissue mucocytes discharge hydroscopic mucin into the seawater directly overlying the coral, creating an instantaneous large-volume expansion of mucus over a large surface area of coral tissue. This coral surface mucus layer has been observed to serve as a medium for nutrient assimilation, and is hypothesized to physically protect coral from chemical exposure by increasing the thickness of uncontaminated mucus in direct contact with the coral [13]. These preliminary results in conjunction with the observed increases in gene transcript expression for xenobiotic detoxification mechanisms are suggestive of both a physical and metabolic response by the coral animal to protect the holobiont in a chemical stressor exposure.

### A hypothetical scenario for RDX-induced energy loss in the holobiont

The critical role of zooxanthellae photosynthesis in generating energy within the coral holobiont is well described [15, 54]. Preliminary results indicate that holobiont bioaccumulation of RDX (Figure 2) did not affect zooxanthellae density in the *A. formosa* holobiont (Figure 7). RDX did however decrease transcript expression for genes involved in carbon fixation from CO_2_ (carbonic anhydrase, AAM94169 and AAW79301, Additional file 2: Table S5), photosynthesis [photosystem II protein (CAI58622, Additional file 2: Table S6), chloroplast light harvesting complex protein (AAW79366, Figure 5) and major facilitator superfamily (AAU87579, Figure 5)], and carbohydrate metabolism (pyruvate kinase, XP_001700637, Figure 5). Consistent with a decreased potential for photosynthetic energy production by zooxanthellae, coral animals decreased transcriptional expression of genes involved large 6 carbon carbohydrate metabolism (UDP-glucose 6-dehydrogenase, XP_971267, Figure 5 and 6-phosphogluconate dehydrogenase XP_001627413, Additional file 2: Table S6) in favor of increased expression for metabolism of smaller 2 and 3 carbon substrates in glycolysis (phosphoenolpyruvate carboxykinase 1, XP_001179448, Figure 5 and enolase 1 alpha, XP_001632906, Additional file 2: Table S6) and the citric acid cycle (citrate synthase, XP_001641037, Additional file 2: Table S6). The transcriptional network generated for combined coral-zooxanthellae carbohydrate and energy metabolism showed strong correlations between the decreased expression of photosynthetic genes in zooxanthellae and increased transcript expression for genes involved in glycolysis, the citric acid cycle and the electron transport chain (Figure 6). Although it is unlikely that these correlative network connections represent canonical feedback in metabolism among coral and zooxanthellae, they do indicate that the coral animal has highly sensitive and integrated transcriptional responses to the energetic potential of the zooxanthellae.

## Conclusions

Our results indicate differential sensitivities and responses to the emerging marine pollutant RDX among species comprising the *A. formosa* coral holobiont. The coral animal had the most sensitive transcriptional response to RDX bioaccumulation (Figure 4) including increased expression of genes involved in xenobiotic metabolism such as phase I and II xenobiotic detoxification pathways, cytochrome P450s and UDP glucuronosyltransferase 2 family enzyme, respectively (Figure 5) at the lowest RDX exposure level. These results in addition to preliminary data suggesting increased mucus production (Figure 7) as a physical barrier to RDX exposure indicate that the coral animal may provide a sentinel response to protect the coral holobiont against chemical stressors. As RDX exposure concentrations/bioaccumulation increased, the proportion of differentially expressed transcripts from zooxanthellae dominated the holobiont transcriptional response (Figure 4). Transcriptional network (Figure 6) and metabolic pathway analyses (Additional file 2: Table S6) suggest that the coral holobiont may have reduced potential for carbon fixation and energy production via photosynthesis in zooxanthellae. Observed compensatory increases in expression of glycolytic, citric acid cycle and electron transport pathways were observed likely to sustain energy (ATP) needs in the coral animal. These observations underscore the potential for complex integrated responses that occur among species comprising the coral holobiont and highlight the need to understand holobiont-species interactions to accurately ssess stressor impacts.

### Supporting information

**Supplemental text:** available in the linked PDF document.

**Supplemental tables:** available in the linked Excel spreadsheet.

## Electronic supplementary material

Additional file 1: **Supplemental Text.** (DOCX 86 KB)

Additional file 2: **Supplemental Tables.** (XLSX 3 MB)
