# Supplementary material for: Coral-zooxanthellae meta-transcriptomics reveals integrated response to pollutant stress
Source: BMC Genomics. 2014 Jul 12;15(1):591. doi: 10.1186/1471-2164-15-591 (PMC4117956; doi:10.1186/1471-2164-15-591)
Supplement: Supplementary file 1 — Additional file 1: Supplemental Text. (DOCX 86 KB) [file 12864_2014_6291_MOESM1_ESM.docx]

**(Additional File 1 - Supplemental Text)**

**Title: Coral-Zooxanthellae Meta-Transcriptomics Reveals Integrated Response to Pollutant Stress**

**Short Title:** Coral Meta-Transcriptomics Reveal Pollutant Stress

**Authors:** Kurt A. Gust^1*^, Fares Z. Najar^2^, Tanwir Habib^3^, Guilherme R. Lotufo^1^, Alan M. Piggot^4,5^, Bruce W. Fouke^4,6^, Jennifer G. Laird^1^, Mitchell S. Wilbanks^1^, Arun Rawat^7^, Karl J. Indest^1^, Bruce A. Roe^4^, Edward J. Perkins^1^

^1^US Army Engineer Research and Development Center, Environmental Laboratory, Vicksburg, MS, 39180, USA. [kurt.a.gust@usace.army.mil](mailto:kurt.a.gust@usace.army.mil), [Guilherme.Lotufo@usace.army.mil](mailto:Guilherme.Lotufo@usace.army.mil), [Jennifer.G.Laird@usace.army.mil](mailto:Jennifer.G.Laird@usace.army.mil), [Mitchell.S.Wilbanks@usace.army.mil](mailto:Mitchell.S.Wilbanks@usace.army.mil), [Karl.J.Indest@usace.army.mil](mailto:Karl.J.Indest@usace.army.mil), [Edward.J.Perkins@usace.army.mil](mailto:Edward.J.Perkins@usace.army.mil)

^2^University of Oklahoma, Advanced Center for Genome Technology, Norman, OK, 73019, USA. [fznajar@ou.edu](mailto:fznajar@ou.edu), [broe@ou.edu](mailto:broe@ou.edu)

^3^Badger Technical Services, San Antonio, TX, 71286, USA. [tanwir.habib@usace.army.mil](mailto:tanwir.habib@usace.army.mil)

^4^University of Illinois, Department of Geology, Urbana-Champaign, IL, 31801, USA.

^5^University of Miami, Division of Marine Geology and Geophysics, Miami, FL 33149, USA. [apiggot@rsmas.miami.edu](mailto:apiggot@rsmas.miami.edu)

^6^University of Illinois, Institute for Genomic Biology, Urbana-Champaign, IL, 31801, USA. [fouke@illinois.edu](mailto:fouke@illinois.edu)

^7^Translational Genomics Research Institute, Phoenix, AZ, 85004, USA. [rawat.arun@gmail.com](mailto:rawat.arun@gmail.com)

*** Corresponding Authors:**

Kurt Gust - [kurt.a.gust@usace.army.mil](mailto:kurt.a.gust@usace.army.mil)

**MATERIALS AND METHODS**

***Coral Acquisition and Processing***

*Acropora sp.* fragments were shipped to the US Army Engineer Research and Development Center (ERDC; <http://www.erdc.usace.army.mil>) in Vicksburg, Mississippi where exposure experiments were conducted. Individual fragments were placed in sealed plastic bags half filled with seawater and pure O_2_, respectively, and shipped via Delta Airlines overnight delivery of living animals service (<http://www.delta.com>). Upon arrival at ERDC, the corals were handled as directed in the instructions provided by Oceans, Reefs and Aquaria Company (ORA, Harbor Branch Oceanographic Institution, Ft. Pierce, FL) to acclimate corals to the receiving aquaria. Briefly, the coral bags were floated in the receiving aquaria for 1 hour to achieve temperature equilibrium. Next, each coral fragment was placed in an individual beaker containing 50% water from the shipping bag and 50% water from the receiving aquarium and allowed to acclimate for 4 hours. The corals were then placed in aquaria designed and licensed for controlled experimentation of exposure to 1,3,5-trinitro-1,3,5 triazine (RDX) where the coral fragments were allowed to acclimate for 24h prior to addition of RDX.

***Experimental Design, Statistical Independence & Dose-Response Relationships***

During peer review, there was concern about the ability to achieve independence among biological replicates within the experimental design used in this study. It would have been preferable from a design perspective to have each coral fragment housed in an individual aquarium, however the cost of the elaborate coral husbandry equipment and the tremendous footprint required to execute such an experiment was prohibitive.

A primary concern for the peer reviewers was that the samples were “pseudo-replicated” and therefore the samples were not independent violating a critical assumption of the ANOVA used to analyze and draw inferences from the experiment. With respect to the reviewers’ concerns, we argue that variation in transcript expression responses in individuals (biological replicates) was not dependent upon the aquaria they were exposed in and had marginal impact on chemical effects observed across different aquaria indicating that the experimental design did not invalidate the results described in the paper. The logic and observations in support of this argument are provided in the following text:

First, we would like to point out a very important distinction between spatiotemporal independence and statistical independence. As stated by Schank and Koehnle (2009) in a critique on the concept of pseudo-replication, “… *independence is not mathematically related to either proximity or isolation*. Stated simply, two events are statistically independent if and only if the occurrence of one makes the other neither more nor less probable. Numerous factors can influence statistical independence such as spatiotemporal proximity, but these factors should not be confused with the definition of statistical independence.”

Further, they state … “Whether or not spatial relationships, boundaries, or physical connectedness generates statistical dependencies is an empirical question. Our central argument is that statistical dependencies can be detected with appropriate multilevel models, control of physical conditions, or replication of studies.”

In the following text, we speak to the three criteria listed above to demonstrate due diligence in describing the state of statistical independence and sources of variation in our study:

1. *Statistical dependencies can be detected with appropriate multilevel models*: As recommended by Schank and Koehnle (2009)^1^, we sought to provide empirical evidence of statistical independence among coral samples in our study and specifically demonstrate that RDX was the primary source of variation across treatment groups. Using a concept fundamental to the toxicological sciences, the dose-response relationship, we now demonstrate that RDX was the fundamental driver of (A.) changes in overall transcript expression, as well as for (B.) critical biological processes / pathways affected in xenobiotic exposures. If non-independent responses or “batch effects” occurred within aquaria, then random, punctuated or divergent responses across the exposure concentration range would be expected. Stated explicitly, if dependency among coral fragments was the primary source of variation across experimental treatments, then few if any dose-response relationships would be expected in the RDX exposures.

(A.) Overall changes in transcript expression (Dose-response relationships): As demonstrated in Figure 3 of the manuscript, a dose-response relationship of increasing overall transcript expression in response to increasing RDX concentration was observed. Further, a dose-response relationship was observed in the relative expression of transcripts within the holobiont where the relative proportion of transcripts differentially expressed by the zooxanthallae increased with RDX exposure concentration (Figure 4). Further, that dose-response relationship in the zooxanthellae showed decreased expression of transcripts with increasing RDX exposure concentration (Figure 4 and Supplemental Figure 1, below), demonstrating negative effects of RDX on transcript expression for multiple metabolic pathways in zooxanthellae.

45 Unique Gene Transcripts Total

Supplemental Figure 1 (in support of supplemental text). Expression for all transcripts having significant differential expression in the zooxanthellae in response to RDX exposure.

(B.) Critical biological processes / pathways expected to be affected in xenobiotic exposures (Dose-Response Relationships): Dose-response relationships were also found for important biological and metabolic processes in the coral holobiont. For example, in the zooxanthellae, differentially expressed gene transcripts involved in supporting photosynthesis had a negative dose- response relationship with increasing RDX concentrations (Supplemental Figure 2, below).

Supplemental Figure 2 (in support of supplemental text). Expression for gene transcripts derived from zooxanthellae that are known to be involved in photosynthetic systems / function that had significant differential expression in response to RDX exposure.

In examining functional responses within the coral animal, a positive dose-response relationship with increasing RDX concentrations was observed for multiple gene transcripts involved in both Phase I and Phase II xenobiotic detoxification mechanisms (Supplemental Figure 3, below).

The evidence we have described above indicates the prevalence of RDX dose-response relationships at many levels of our transcript expression investigations. This evidence demonstrates that even if statistical dependencies did exist within exposure aquaria, the predominant source of variation within our experiments was the RDX exposure relative to any interactions between coral fragments. There is no denying that there was physical independence between aquaria, however if interactive effects within tanks was the primary driver of changes in transcription expression, dose-responses (especially dose-responses indicative of xenobiotic exposure) would not be expected.

Supplemental Figure 3 (in support of supplemental text). Expression for gene transcripts derived from the coral animal known to code for proteins involved in xenobiotic detoxification mechanisms that had significant differential expression in response to RDX exposure.

2. *Control of physical conditions:*  As a means to eliminate dependencies among samples we took the following steps. 1. We carefully controlled and monitored the physical conditions within exposure chambers to assure uniformity in water quality, temperature, and lighting conditions within and among experimental chambers (Table S2). Additionally, we carefully monitored the RDX exposure levels in each treatment using analytical chemistry to empirically establish exposure levels daily throughout the exposure assays (Table S2).

3. *Replication of studies:* Replication of studies and testing of hypotheses proposed in the literature are critical to the scientific process. Publication of the observations generated in this work will allow researchers to consider and test specific hypotheses to advance ecotoxicology as well as coral holobiont science. We feel that the hypotheses we’ve generated in this work are relevant and important and should be presented for consideration by the scientific community.

In total, we have carefully considered the implications of statistical independence in our study, and the weight of evidence suggests that if non-independence was occurring among biological replicates, that source of variation was largely subordinate to the relatively strong effects of RDX exposure. A variety of dose-response relationships were observed in both total differential expression of transcripts as well as for functional responses within both the coral animal and the zooxanthellae in the holobiont indicating that the transcriptomic responses were primarily a response to the dependent variable, RDX concentration.

As a final note outside of the realm of statistics, in the field, dependent responses where an effect of RDX on a single individual that can negatively influence a near neighbor within the coral colony is certainly an important adverse effect that should be recognized regarding protection of coral species. Therefore, the responses we observed, whether independent or dependent on one sensitive coral fragment within a batch represents an important observation regarding coral health in the environment.

***Context for Coral Exposure to RDX in the Environment***

Unexploded ordnance (UXO) and discarded military munitions (DMM) in marine environments may pose a risk to the local biota because shell casings may eventually breach through mechanical stress, corrosion, and low-level remedial detonations and release potentially harmful chemicals. However, exposure needs to be evaluated based on spatial considerations and is likely insignificant to most individual receptors at underwater explosive contaminated sites. Sessile organisms, such as corals, face higher exposure likelihood compared to mobile organisms with variable residence time, particularly in the localized areas surrounding a potentially breached shell. Adequate records of measured concentration of explosive compounds in the water surrounding UXO and DMM in tropical areas were not found in the available literature.

***Analytical Chemistry: Exposure Water and RDX-Tissue Residues***

High performance liquid chromatography (HPLC) was used to quantify the RDX concentrations in water samples from each tank as well as in solvent extracts of tissue samples. The HPLC process was performed following the U.S. Environmental Protection Agency SW-486 Method 8330. Analyses were conducted with an Agilent 1100 Series HPLC (Palo Alto, CA) equipped with a Supelco RP-Amide C-16 column and a diode array detector. Sample injection volume was 100 µl with a flow rate of 1 ml per minute and column temperature of 45°C. An isocratic mobile phase consisting of 55% methanol and 45% water was utilized. Absorbance was measured at 230 and 254 nM. Peak identification was based on retention time with spectral analysis confirmation. The laboratory reporting limit for all analytes was 0.5 µmol/L (~ 0.1 mg/L) for water samples, and 5 µmol/kg (~ 1 mg/kg) for tissue samples. Recovery of RDX from tissue extracts ranged from 90 to 98%.

***Reverse-transcriptase, quantitative polymerase chain reaction (RT-qPCR)***

Eight hundred nanograms of DNase-treated total RNA for each biological replicate was reverse transcribed using Random Primers (Invitrogen, Carlsbad, CA) in a SuperScript III (Invitrogen) catalyzed reaction. RT-qPCR was performed using an Applied Biosystems (ABI) Prism 7900HT Sequence Detection System (Applied Biosystems, Foster City, CA) with each reaction mixture containing ABI SYBR^®^ Green Master Mix, target primers at a concentration of 5µM and approximately 60 ng of cDNA product. Primer melt curves were conducted after qPCR and data for any primer sets that did not have a single conserved melt peak were discarded.

**REFERENCE**

Schank JC, Koehnle TJ. Pseudoreplication is a pseudoproblem. J Comp Psychol. 2009 Nov;123(4):421-33. doi: 10.1037/a0013579.
